# Molecular, Genetic and Agronomic Approaches to Utilizing Pulses as Cover Crops and Green Manure into Cropping Systems

**DOI:** 10.3390/ijms18061202

**Published:** 2017-06-05

**Authors:** Eleni Tani, Eleni Abraham, Demosthenis Chachalis, Ilias Travlos

**Affiliations:** 1 Laboratory of Plant Breeding and Biometry, Department of Crop Science, Agricultural University of Athens, IeraOdos 75, Athens 11855, Greece; 2 Laboratory of Range Science, Faculty of Agriculture, Forestry and Natural Environment, Aristotle University of Thessaloniki, Thessaloniki 54124, Greece; eabraham@for.auth.gr; 3 Laboratory of Weed Science, BenakiPhytopathological Institute, S. Delta 8, Athens 14561, Greece; d.chachalis@bpi.gr; 4 Laboratory of Agronomy, Department of Crop Science, Agricultural University of Athens, IeraOdos 75, Athens 11855, Greece; travlos@aua.gr

**Keywords:** cover crops, grain legumes, cowpea, faba bean, pea

## Abstract

Cover crops constitute one of the most promising agronomic practices towards a more sustainable agriculture. Their beneficial effects on main crops, soil and environment are many and various, while risks and disadvantages may also appear. Several legumes show a high potential but further research is required in order to suggest the optimal legume cover crops for each case in terms of their productivity and ability to suppress weeds. The additional cost associated with cover crops should also be addressed and in this context the use of grain legumes such as cowpea, faba bean and pea could be of high interest. Some of the aspects of these grain legumes as far as their use as cover crops, their genetic diversity and their breeding using conventional and molecular approaches are discussed in the present review. The specific species seem to have a high potential for use as cover crops, especially if their noticeable genetic diversity is exploited and their breeding focuses on several desirable traits.

## 1. Introduction

Due to the present-day large-scale reliance on agrochemicals and intense soil exploitation, several agronomic practices have recently grained increased attention and extensive study. Cover crops are among these practices. By the term “cover crops”, we often mean crops that are implemented between two main crops and are known to provide various ecological services in agro-ecosystems [1]. Moreover, those crops that are sown between the rows of perennial crops (trees, vines, etc.) can be also characterized as cover crops [2]. According to the Soil Science Society of America, cover crops can be also defined as “close-growing crops (not grown for market purposes) that provide soil protection, and soil improvement (when plowed under and incorporated into the soil), frequently referred to as green manure crops, grown in between periods of normal crop production”.

Cover crops can be leguminous or non-leguminous, depending on the specific objective set; primarily, leguminous cover crops are used as a source of nitrogen (N) for the following cash crop [3] and therefore reducing the need for N in fertilizers [4], as compared to the non-leguminous crops mainly used to reduce NO_3_ leaching and erosion [5]. The aim of the present review is to briefly report the advantages and disadvantages of the use of cover crops into cropping systems and finally to focus on the main grain legumes that are used as cover crops. Additionally, the major breeding efforts and molecular studies on the traits important for their use as cover crops will be presented. This would be a helpful tool for anyone that works on pulses generally, i.e., breeding, cultivation, etc., and especially as cover crop breeders.

## 2. Cover Crops, Advantages and Disadvantages

Organic agriculture cannot be sustainable without soil protection, biological pest and weed control and avoidance of soil erosion [6,7]. Cover crops are certainly considered to be among the most common and compatible practices with organic farming [8].

Sowing cover crops enhances microbial activity and improves soil structure. Moreover, especially in orchards and vineyards, it can promote a long-term crop productivity without the risks of competitive weeds, low organic matter and soil erosion [9]. Furthermore, cover crop vegetation contributes to overall biodiversity, provides an increased aesthetical value for eco-touristic purposes and favors ecosystem services such as pollination or pest-control [10].

Cover crops are utilized in perennial crops such as vineyards throughout the world for a variety of reasons [11,12]. Aside from their primary role in soil stabilization, cover crops are used with varied success to regulate vegetative growth and vigor [13,14]. Cover crop competition that improves vine canopy characteristics and berry environment can indirectly improve berry composition [15,16]. Cover cropped inter-rows are widely used in US and temperate European vineyards and are being evaluated in countries such as China [12,17,18]. Due to all these benefits, use of cover crops has lately become a common practice in many regions [19,20,21]. Protection against soil erosion, reduction of nutrient losses, improvement of soil and water quality, and reduction of weeds and pests have been recognized by many authors among the most beneficial effects of cover crops [9,22,23]. Furthermore, adding nitrogen (N) fixing legume species as a cover crop can improve N nutrition of the succeeding main crop and increase the soil N organic pool [24].

Despite the above-mentioned advantages, cover crops are not widely used by farmers, mainly due to additional costs and labor requirements [1]. Moreover, cover crop effects on crop productivity, crop nutrition, or weed control are often variable and depend on cover crop species, soil type, and climate [24]. Cover crops can be adapted in a wide range of soil and climate conditions. However, proper management and selection of cover crops are essential to maximize the advantages and minimize drawbacks. Cover crop selection should be certainly targeted to the specific needs of the farmer and the agro-ecosystem. For instance, in Mediterranean regions (and not only them), soil moisture is a crucial factor for the establishment of cover crops and sowing in autumn–winter is strongly suggested. In poor soils of low fertility, the selection of some legumes as cover crops could result in significant benefits, while in sandy soils of bad structure some *Poaceae* species could be a very good choice [2].

**Figure 1:**
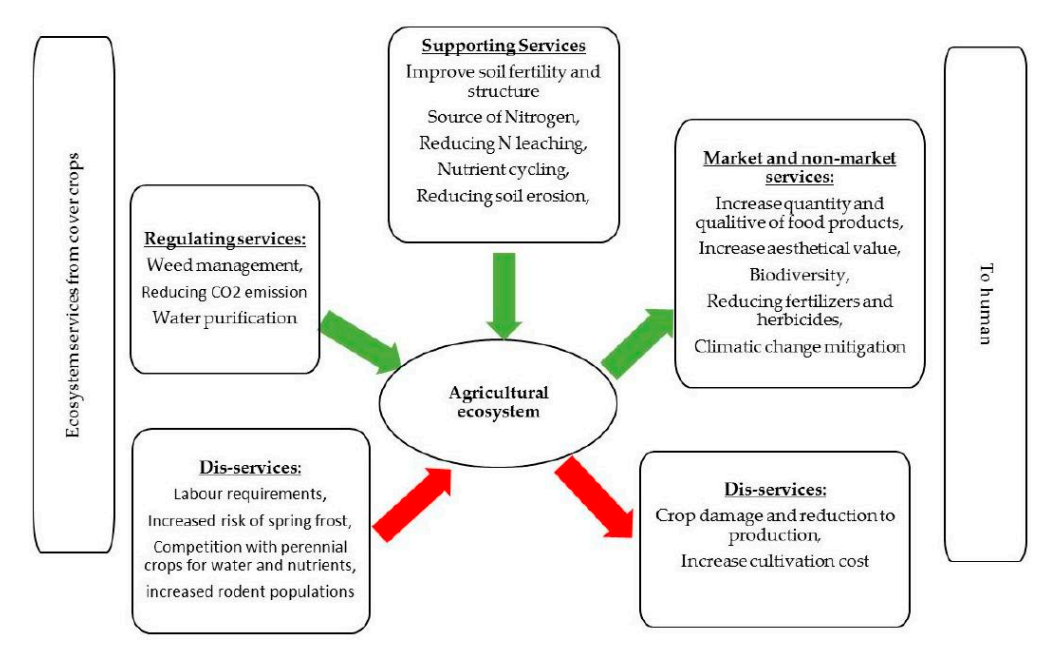
Ecosystem services from the cover crops to agricultural ecosystem and to human.

Potential disadvantages include not only competition with crops for water and nutrients but also cost of establishment, need for regular maintenance, increased risk of spring frost, and crop damage from increased rodent populations [11,25,26]. To date, most research on cover crops has focused on their effects on water quality and N dynamics [22,24] or on the choice of plant species [23] and management options [24,27] (e.g., sowing and killing techniques and dates). The advantages and disadvantages of cover crops are summarized in Figure 1.

## 3. Focus on Leguminous Cover Crops

Several legume species have been tested as cover crops. Species such as *Lotus corniculatus*, *Trifolium repens* and *T. pratense* but also grain legumes such as *Pisum sativum*, *Vicia faba* and *Vigna unguiculata* can establish well and have high cover scores even for the second year [10,28]. It has to be noted that the low C:N ratio of legumes is highly desirable as long as it results in the rapid decomposition of their residues [29]. For instance, C:N ratios for red clover and hairy vetch are considered to be around 15:1 and are characterized as optimum values [30].

The amount of atmospheric N fixed by legumes varies greatly depending on type, inoculation effectiveness, and soil moisture and temperature [31]. Estimations of N fixation by vetch (*Vicia* spp.) ranged from 50 to over 220 kg/ha, and for strawberry clover (*Trifolium fragiferum* L.) from 100 to over 330 kg/ha [18]. The impact of legume cover crops on productivity is known to be strongly related to the amount of nitrogen fertilization [32,33,34]. On the other hand, leguminous cover crops often lack persistence but may also supply more nitrogen than desired in an already high vigor crop [35].

Previous studies have evaluated the performance of several cover crops in organic vineyards in California [36,37]. Common vetch (*Vicia sativa* L.), annual ryegrass (*Lolium multiflorum* Lamarck), wildoat (*Avena fatua* L.), and berseem clover (*Trifolium alexandrinum* L.) ensured a high soil coverage, while species such as *Bromus* spp. annual ryegrass (*L. multiflorum*), blue wildrye (*Elymus glaucus* Buckely), wildoat (*A. fatua*), rye (*S. cereale*), crimson clover (*T. incarnatum* L.), subterranean clover (*T. sub-terraneum* L.), and strawberry clover (*T. fragiferum*) revealed a high regeneration capacity [36].

## 4. Regulating Services from the Leguminous Cover Crops to Agro-Ecosystems

### 4.1. Weed Management

As stated by Travlos (2010), to optimize the use of cover crops, it is necessary to avoid (or minimize) interference (competition and allelopathy) of the cover crops and the main crop [2]. Leguminous cover crops (hairy vetch) could reduce weed density in organic no-till corn by at least 50%, with annual weeds being affected more than perennials. Total weed biomass was reduced 31–94% (depending on the site and the year) compared with no-cover plots [38]. However, weed suppression effects of legumes cover crop residue decrease with time, after cover crop residue decomposition, and therefore use of synthetic herbicides are necessary to achieve weed control through full seasons and optimum yields in the subsequent market crop (corn) [39].

There are a number of mechanisms responsible for the effect of cover crops on weeds. The living cover crop can significantly reduce the extent of light penetration with severe physiological sequences to soil weed seed bank [40]. Cover crops may alter soil conditions or even release allelochemicals and consequently restrict weed seed germination and seedling emergence [39,40,41]. Additionally, many legumes (bean, pea, and vetch) produce quinolizidine alkaloids that may be responsible for allelopathy, while in other legumes such as velvet bean (*Mucuna deeringiana*) l-3,4-dihydroxyphenylalanine (L-DOPA), an intermediate of many alkaloids, was determined to be the allelochemical [42].

The additional cost associated with cover crops should be covered by increased yield and/or reduced herbicide cost. Several legumes show a high potential but further research is required in order to suggest the optimal legume cover crops for each region in terms of their productivity and ability to suppress weeds. Several legumes as living cover crops could contribute to the reduction of the weed seed bank in soils as long as they do not compete with the main crop [43].

### 4.2. Reducing CO_2_ Emission

This attribute is mainly achieved by reduced tillage or direct seeding of the leguminous cover crops. For winter sowing legumes that are grown under rainfed Mediterranean conditions, positive outcomes of the no-tillage systems have been documented, as in faba bean [44]. Additionally, in a long-term experiment (an 11-year study period), the no-tillage faba bean system resulted to similar yields, biological nitrogen fixation (BNF) and percent of N derived from the atmosphere (NdfA) compared to the conventional system [45]. Minimum tillage could be successfully used either on wetter cropping systems [44] or in semiarid regions, as demonstrated in southern Italy [46].

Life cycle assessment (LCA) was performed to compare a conventional system (regular use of the plow) with a strict direct seeding system (no-till); it was shown a reduction of 8% per unit area and 12% per unit of product, in the no-tillage system [47]. Direct seeding would result in significant energy saving of up to about 90% of the diesel fuel that was used for mouldboard plowing, seedbed preparation, and sowing in a loose soil husbandry system [48].

In a comprehensive study, a comparison of various crops in Swiss lowlands, in terms of the environmental impacts was performed [49]. Grain legumes had a clearly lower energy demand per unit area (13–14 vs. 20–22 GJ/ha, in cereals) andlower global warming potential (3209–3217 vs. 4126–5796 kg CO_2_-eq./(ha), in cereals). Additionally, in cropping systems (such as potatoes andmaize) where more intensive machinery operations are used, grain legumes have an even lower environmental impact profile.

Direct seeding of leguminous cover crops poses significant challenges as follows:

(a) In almost all cases, the no-till systems are highly dependent on herbicide use such as the non-selective glyphosate or other contact herbicides that would have higher environmental impact such as terrestrial ecotoxicity.
(b) Yields are often maintained with higher mineral nitrogen input due to tillage operations especially in the first years of continuous use of reduced tillage in temperate climates [50].
(c) Control of weeds (annual or perennial) tends to depend on tillage operations.
(d) Launch of appropriate soil conservation technologies similar to what has been successfully used with soybean for decades in mainstream agriculture [51].
(e) Integration of mulch seeding or direct seeding of leguminous cover crops in systems where grasses have been used as a superior mulch (e.g., oats for annual weeds control due to competition and allelopathy).

## 5. The Most Common Grain Legumes as Cover Crops

The main grain legumes that have been used as cover crop were reported by Fageria (2007) [28]. One of the most common of the tropical ones is cowpea (*Vigna unguiculata* L. Walp.) and of the temperate region is faba bean (*Vicia faba* L.) and pea (*Pisum sativum* L.). The uses of these grain legumes as cover crops (Table 1), their breeding and the molecular approaches will be reviewed below.

### 5.1. Cowpea (Vigna unguiculata L. Walp.) as a Cover Crop

Cowpea (*Vigna unguiculata* L. Walp.) is a self- pollinating annual legume, and it is also referred to as southern pea, black-eyed pea, crowder pea, lubia, niebe, coupe or frijole. It is widely used as a grain legume and a major source of dietary protein because it contains about 25–30% protein in the grains and 15–18% protein in its haulms. It is a primary source of income for small holder farmers in Africa, since 85% of dry cowpea production comes from three countries (Niger, Nigeria and Burkina Faso) [52]. It is also used for animal fodder, or as a vegetable [53]. In Africa, tender green leaves as well as green and mature seeds are boiled and eaten. It is also of great importance as a cover crop, a green manure crop, a nitrogen-fixing crop and a crop for preventing soil erosion [54].

Cowpea is considered as a key player of sustainable farming in semiarid lands. In these soils, with sandy loan texture, moderate to low natural fertility and low external inputs, intercropping of grain crops (sorghum and millet) with cowpea is a common practice. Cowpea is a fast growing crop and its bushy growth provides ground cover, weed suppression and protection against soil erosion. It fixes atmospheric N and its soil residues ameliorate soil fertility. In addition, some cowpea varieties suppress the germination of the seeds of *Striga hermonthica*, a major threat of cereals often with devastating effects [55]. *V. unguiculata*is also broadly used as a warm season, nitrogen fixing cover crop, particularly in organic vegetable production systems.

Cowpea carries some traits that make it an excellent warm season cover crop: it is tolerant to heat and drought stress; it grows well in sandy, poor, acidic soils; it produces high biomass; and has a high nitrogen fixation capability [56]. Due to its ability in restoring soil fertility, it is broadly used in crop rotation schemes for succeeding cereal crops [57,58,59].

Nematode and nitrogen management could also be very efficient when using cowpea as a cover crop in organic production systems. Cowpea has been documented to successfully manage plant-parasitic nematodes on basil (*Ocimum basilicum* L.) and Chinese cabbage (*Brassica chinensis* L.) when used as summer cover crop [60]. In vegetable production systems that are of great economic significance worldwide, an adequate weed management is of great importance as few herbicides are registered. As mentioned earlier, cowpea is vigorous, produces large amounts of biomass and can be used to suppress weeds during fallow year in tropical and subtropical regions [61,62]. When cowpea was used as a cover crop for organic production of onion, it contributed to a very high marketable onion yield and satisfactory weed suppression [63]. Cowpea mulches, originated from the residues of killed cowpea, lowered the needs for N requirements of the broccoli cultivation that succeeded and increased significantly its yield compared to conventional, bare soil production. Cowpea mulches also had a great impact on suppressing annual weeds and nematodes [64]. In studies conducted for cabbage and lettuce, cowpea used as a cover crop increased soil nitrate concentration, produced the highest marketable cabbage and lettuce yields and shortened their harvesting time.

It was found that weed suppression when cowpea was used as a cover crop could also be due to allelopathy. Species such as cowpea, sun hemp, and velvet bean can be used during summer periods to suppress weeds and it is likely that these cover crops can have an allelopathic effect on the germination of certain weeds. For example, smooth amaranth germination and plant height were inhibited by ground dried residues of all three cover crops. However, bell pepper and tomato seed germination was also suppressed by cowpea residues [65].

Moreover cowpea used as a cover crop can have a positive effect on the quality of the marketable product. In a sugarcane field, when cowpea was used as a cover crop, there was an increase in sugarcane total sucrose that is recoverable from sugarcane [66].

Cowpea crop has two disadvantages that should be considered when used as cover crops: it is cold sensitive and sensitive to waterlogging. Additionally, cover crops of cowpeas are sometimes attacked by cowpea aphid (*Aphis craccivora*), which could influence the following crop.

#### 5.1.1. Origin and Adaptation

Cowpea is cultivated not only in Africa, but also in Latin America, Southeast Asia (mainly in India) and in the southern United States [53,55]. It is also cultivated to a lesser extent in Oceania, and Southern Europe. Cowpea is considered as one of the most ancient human food sources and its cultivation goes back to ancient West African cereal farming, 5000–6000 years ago [67]. A recent study has demonstrated that cowpea originated from two main areas (West and East of Africa) which were also the first domestication regions. Moreover India is considered as a sub-domestication region of cowpea [68]. All cultivated cowpea varieties belong to *Vigna unguiculata* [69] as well as its close cross-compatible relatives.

Three wild subspecies of *V. unguiculata* (*V. unguiculata* ssp. *dekindtiana*, *V. unguiculata* ssp. *stenophylla* and *V. unguiculata* ssp. *Tenuis*) have been recognized [70]. Several taxonomists have proposed different subspecies and names for some of the cowpea wild relatives. There is a lack of proper classification of the cowpea wild relatives and this creates obstacles to the definition of primary and secondary gene pools for cowpea [70]. A major drawback of the use of cowpea wild relatives in breeding the cultivated cowpea varieties is their small seed-size characteristic. However, this is a desirable trait (as mentioned below) for breeding cowpea varieties for use as a cover crop.

#### 5.1.2. Genetic Diversity

Cowpea is a diploid species (2*n* = 22) with an estimated genome size of 620 Mb [71]. Other warm season legumes, especially mung bean (*Vignaradiata*; [72]), common bean (*Phaseolus vulgaris* L.; [73]) and soybean [74], share a high degree of collinearity with cowpea genome. The exploitation of cowpea genomic resources and their use for genetic improvement has been very recent.

Diverse cowpea germplasm is available from collections all over the world, including the USDA repository in Griffin, GA (USA), the University of California, Riverside, CA (USA), and the National Bureau of Plant Genetic Resources (NBPGR) in New Delhi; however, The International Institute of Tropical Agriculture (IITA) maintains the majority of cowpea genetic resources (about 15,000 accessions of cultivated cowpea and more than 2000 wild relatives). Most of the early efforts have been focused on the genomic scale sequencing and the development and use of molecular marker technologies that have facilitated the identification of diversity in the available germplasm and different sources of genes for several of the desirable traits for cowpea breeding [75,76,77]. The most detailed genomic resources were developed from the African cultivar IT97K-499-35 that is tolerant to most *Stringa* strains [74,78]. Very recently, a whole genome sequence was conducted for 36 diverse cowpea accessions that further supported the development a consensus genetic map containing 37,372 single-nucleotide polymorphism (SNPs) [79]. A very detailed review by [70] has gathered all the recent advances in cowpea genomic resources. They highlight the development of several platforms that facilitate the discovery of genes of interest such as The Cowpea genomics Initiative, The Cowpea Genomics Knowledge Base, etc. Many linkage maps with very high marker density have been developed, thus enabling quantitative Trait Locus (QTL) resolution, map-based cloning, association mapping, and marker-assisted breeding. The big obstacle is that the genetic linkage maps that have been published are not yet aligned with physical cowpea chromosomes due to the fact that cowpea lacks a published reference genome. Thus, there is an ongoing genome sequencing project (Available online: https://www.integratedbreeding.net/126/communities/genomics-crop-info/agricultural-genomics/genome-sequencing/cowpea) undertaken by a team of UC Riverside scientists. Finally, the synteny and co-linearity that has been reported among cowpea, soybean and mung bean [72,73,74] can be utilized in marker-assisted breeding programs as well.

#### 5.1.3. Important Traits for Breeding and Molecular Approaches

Based on several studies, an ideal cowpea cultivar for use as cover crop should have the following characteristics: production of abundant biomass, photoperiod sensitivity, vigorous shoot type to suppress weeds, reduced pod shattering and small seeds to facilitate harvest and planting, and strong resistance to nematodes and other pathogens (such as *Fusarium* wilt) [80,81]. First, photoperiod sensitive cowpea used as a cover crop produces much more biomass and fixes much more nitrogen because, under long day lengths, photoperiod sensitivity prevents the transition to reproductive growth that leads to a decline in biomass production and a lower nitrogen fixation. However, photoperiod sensitive cultivars could display slow early growth and development that may be advantageous to weed expansion.

On the other hand, photosensitive cowpea cultivars grown under environments where day lengths is under 12.5 h can flower early, have an early vigor and show extreme dwarf morphology. Cowpea genotypes with early vigor are of great importance in order to successfully suppress weeds. Interestingly, a complete association has been observed between dwarfing and photosensitivity and is controlled by a monogenic, recessive gene [82]. When cowpea is grown as a minor crop in a cereal-based cropping system, it should have a short cycle to fit within the cropping system.

Efforts for breeding cowpea for resistance to nematodes and high biomass have been made by conventional breeding a while ago. UCR-779, a landrace with high biomass production and very aggressive growth but with susceptibility to nematodes had been crossed with two breeding lines from IITA that produce high biomass and have very strong resistance to root-knot nematodes [83], leading to nematode resistant and high biomass breeding lines which were also photoperiod sensitive and with no shattering pods. Recently, a major QTL conferring resistance to root-knot nematodes has been mapped [84]. Moreover, according to [85], lines mapped a *Fot*race3 resistance locus (*Fot3-1*) to *Fusarium oxysporum* f.sp. *tracheiphilum.* The development of cowpea varieties with healthy root systems could be achieved in the near future by pyramiding QTLs for resistance to both root-knot nematodes and *Fusarium* wilt into breeding cultivars.

The genetic variability of cowpea in terms of effective nodulation and nitrogen fixation has been thoroughly discussed by [77]. In addition, a project started in 2016 is underway for breeding cowpea towards enhanced nitrogen fixation in order to improve soil nitrogen and phosphorus content at the Institute of Agricultural Research for Development. According to the description of the project their final goal is to select cowpea with high biomass and nodulation capacity and measure the residual effects of cowpea on maize yields on field trials.

The identification of new desirable traits in cowpea germplasm, with the aid of the new genotyping has been more accurate and the results can be easily incorporated into breeding programs. Souleymane et al. [86] identified a good source of resistance genes against aphid from a cowpea wild relative, TVNu 1158 and also by screening 105 cowpea cultivars, came across the cultivar IT97K-556-6 with tolerance to aphids, in which two resistance loci were mapped [87]. However, no other insect-resistance factor has been discovered that provides substantial resistance due to low heritability under field conditions. This problem could be solved by finding resistance genes outside the cowpea genome and transferring them into cowpea through biotechnological approaches.

Marker assisted breeding (MAS) was successfully applied in creating cowpea lines resistant to *Striga gesnerioides*. Currently, sequence confirmed amplified region (SCAR) markers were found suitable for identifying most of the major race specific resistance genes to S. *gesnerioides* and they were utilized in MAS breeding [88,89]. Besides *Striga*, an restriction fragment length polymorphism (RFLP) marker was found to be associated with the rust caused by *Uromyces vignae* and it was converted to a SCAR marker [90].

Wild relatives of cowpea are sources of many agronomical traits [91]. However, the inherent pod shattering trait of these wild relatives is an undesirable character. Recently a QTL was identified [92] in yard long bean (a legume crop that has been domesticated from wild cowpea) responsible for pod shattering trait. Thus, genomic tools can help in eliminating undesirable traits from improved cultivars.

### 5.2. Faba Bean (Vicia faba L.) as a Cover Crop

*Vicia faba* L. (Fava bean, Faba bean, Broad bean, Horse bean, and Windsor bean) is an annual grain legume (pulse) that is mainly used in human diets because of the nutritive value of its seeds [93] as well in animal feeding as forage, silage, and hay [94]. The replacement of soybean meal by locally produced faba bean could be beneficial for the livestock farming [95]. Furthermore, faba bean is used as a winter or spring cover crop mainly in temperate regions [28] and could be an excellent green manure [96].

The main importance of faba bean as cover crop is to sustain soil fertility and productivity through to its ability to fix nitrogen and to add it into the soil [95]. Faba bean is capable of fixing more nitrogen than the other legumes species [28] and maintain high rates of fixation even under high amounts of available N in soil [97]. Additionally, it accumulates more C into the soil compared to other winter cover crops such as white clover (*Trifoliumvrepens*), purple vetch (*Viciavbenghalensisv* L.) and others [98]. Its use as a cover crop in a Chardonnay/99 Richter vineyard in South Africa improved soil organic matter content and N availability [99]. Furthermore, the faba bean cover crops increased the potato production about 15% more that the common vetch and winter wheat and contributed to the reduction of N application expenses [100]. However, tomato fruits were less firm when tomato followed faba bean, probably because of the higher N accumulation [101]. Regarding soil fertility, it enhances the phosphorus (P) uptake in cereals cultivations [102]. Its role as cover crop could also be the pest management. It contributes in a Citrus orchard to enhance the population of predacious mite (*Euseius tularensis*), a generalist predator that control the citrus thrips (*Scirtothrips citri*) and citrus red mite (*Panonychus citri*) in California [103].

On the other hand, as it is a weak weed competitor, it does not effectively prevent the growth either of winter-growing or summer-growing weeds in vineyards of South Africa [104,105]. However, faba bean cover crop mix with oak and other legumes effectively and economically controlled the weeds in organic vegetable systems [106]. Additionally, it was less efficient as cover crop than other legumes and grasses in soil erosion prevention in a Sicilian vineyard [107]. Generally, as the cover crops increase the soil organic matter could prevent soil erosion [108,109] and furthermore sequester the atmospheric CO_2_ mitigating climate changes. However, according to [110] the increase of soil C above a threshold could contribute to ecosystem degradation through higher C loss in the system. In this regard, the loss of C was higher in plots covered with faba bean and vetch compared to plots with conventional tillage in a Sicilian eroding vineyards [111].

#### 5.2.1. Origin and Adaptation

The origin of the species is unclear. The Near East [112] and Central Asia [113] are referred as possible areas, while recent archeological findings placed its origin in southwestern Asia [114]. Nevertheless, it is one of the oldest cultivations in the world. It was found in the late 10th millennium Before Present (B.P.) at Tell el-Kerkh, in northwest Syria [114] and it spread in Europe, North Africa and China. In earlier times, it was introduced to South America and Australia. It has to be noted that the ancient Greeks used faba bean as green manure around 300 Before Christ (BC) [28]. Nowadays, it is among the most important annual legumes worldwide after soybean (*Glycine max* L.), bean (*Phaseolus vulgaris* L.) and pea (*Pisumsativum* L.) [94]. It is cultivated in 60 countries covering an area of about 2.5 million hectares [52] while China is the major producer followed by Ethiopia. In Europe, it is cultivated in France, Spain, UK and Greece.

It grows better in cool and moist climatic conditions (precipitation of 650 to 1000 mm) [115,116]. It tolerates winter temperatures of −10 to −15 °C depending on the cultivar. Regarding the soil, it is cultivated on all types of soils, but better on rich loams [117]. It grows in neutral to alkaline soils [118] while it grows better than all other legumes in acid soils [119]. On the other hand, it is less tolerant to drought stress compared to other pulses [117]. Nowadays, its cultivation is broadly distributed from the sea level to above 3000 m [120].

#### 5.2.2. Genetic Diversity

*V. faba* is a diploid (2*n* = 2× = 12) facultative cross-pollinated legume with a ~13,000 Mb genome, one of the largest among the legumes (25 times larger than that of *Medicago truncatula*) [121]. The cross-pollination is depending on the presence of insects with honey bees to be the main pollinators [117]. Its large genome is probably a reason for the limiting research on its genetics and genomics. Additionally, its wild progenitor is unknown and it is not hybridized with any of the other *Vicia*species [122]. This means that the only genetic resources for its breeding are the cultivated species. It is classified in four groups based on the seeds size: (1) major, 1.0–2.0 g/seed; (2) equina, 0.6–1.0 g/seed; (3) minor, 0.4–0.6 g/seed; and (4) paucijuga, 0.31–0.40 g/seed [112] and based on region of adaptation in winter, spring and Mediterranean types. The small-seeded germplasm (minor, equina, and paucijuga) is mainly used as animal feed, cover crops, and green manures.

The genetic diversity of its germplasm was study using RFLP molecular markers in different inbred lines and cultivars from Asia and Europe [123], random amplified polymorphic DNA **(**RAPD) in European and Mediterranean type germplasm[124], inter-simple sequence repeat (ISSRs) in lines and accessions from Africa, Asia and Europe [125] and in Greek local population [126], amplified fragment length polymorphism **(**AFLPs) in inbred lines from Asian, European and North African origin [127], in winter [128] and spring [129] cultivars, landraces and accessions from China, Asia, Africa and Europe. Generally, a relatively high level of diversity was recorded and the germplasm pool of faba bean from different geographic regions was clearly discriminated.

#### 5.2.3. Important Traits for Breeding

The importance of a species as cover crop is related to its level of tolerance to biotic and abiotic stresses, the speed of establishment and growth in order to compete weeds and to prevent soil erosion and its ability for N fixation. All the above traits comprise substantial subjects for faba bean’s breeding programs. High leaf area (LA), low specific leaf area (SLA) and low leaf nitrogen content (LNC) are few leaf functional traits desirable for a cover crop [130].

There are many faba bean collections worldwide, which are extensively reported by Duc et al. [131], and have been mainly evaluated for resistance to diseases.

A wide range of biotic stresses influences faba bean and different levels of resistance among its germplasm have been reported [132]. The most important among them is the fungal diseases chocolate spot (*Botrytis fabae*), ascochyta blight (*Ascochyta fabae*), rust (*Uromycesviciae-fabae*) and the parasite weed crenate broomrape (*Orobanche crenata* Forsk.) [120]. Despite the fact that much work has been done on improvement of resistance to biotic stresses, in some cases, the conventional breeding has failed to produce stable resistant cultivars.

The drought resistance of faba bean is relatively low [117]. It grows better under irrigation and withstands water logging better than the other pulses. Breeding for drought resistance is usually indirect through breeding for other traits, in particular grain production. Eco-geographic differentiation that partly related to water availability was reported by Khazaei et al. [133] using the focused identification of germplasm strategy (FIGS). In this regard, the use of germplasm from areas of low water availability could be more efficient in breeding programs aimed at producing germaplasm with enhanced drought resistance.

Winter hardiness is an important trait for its use as winter crop. It should be noted that winter hardiness is also affected by other traits such as resistance to snow cover, to diseases and to water logging [134,135]. There are references for winter hardiness diversity among the germplasm pool of faba bean [135]. Recently registered four winter-hardy fava’s germplasm lines, WH-1 (Reg. No.GP-3, PI 674326), WH-2 (Reg. No. GP-4, PI 674327), WH-3 (Reg.No. GP-5, PI 674328), and WH-4 (Reg. No. GP-6, PI 674329) at the USDA-ARS, Western Regional Plant Introduction Station Pullman, WA for cover crop use. Furthermore, the winter crop performance in contrasting climatic conditions (Continental, Oceanic, and Mediterranean) depended on the cultivar. However, none of the tested cultivars produced elevated yields in all climatic conditions [136]. Finally, differentiated response among faba bean varieties has been recorded under shade [137] and salinity stress [138].

The major problems of its breeding are related to its mix mating system [120]. The released cultivars are either synthetics or inbred lines. Despite the fact that cytoplasmic male sterility has been detected in its germplasm [139] no hybrid cultivar has yet been released. However, traditional landraces are still broadly cultivated in many countries in Europe, Asia and Africa in spite of the existence of modern cultivars [120]. In this regard, the local breeding and synthetic varieties performed better than the formal breeding and inbred line ones respectively in Germany [140], probably due to high genotype × location interaction.

#### 5.2.4. Molecular Approach for Breeding

The genomics and genetic studies of faba bean could be a useful tool to its breeding programs. The molecular markers with the associated genes and/or QTLs that have been studied in faba bean germplasm were extensively reviewed by [141]. The majority of them referred to diseases, mainly to chocolate spot (*Botrytis fabae*), (*Ascochyta fabae*) and rust (*Uromycesviciae-fabae*). Recently, expressed sequence tag (EST) and (SNPs) [142] was used in order to identify two novel QTLs that associated with resistance to ascochyta blight and could be implemented to *V. faba* breeding programs.

The knowledge about faba bean’s genome could be enhanced through comparative genomics with other legume species such as *Medicago truncatula*, *Lotus japonicus* and *Glycine max,* the genome of which extensively has been studied [143]. In this regard, an enhanced map in fava bean was developed by Cruz-Izquierdo et al. [144] using a combination of expressed sequence tag (EST) and other markers that have been described before in order to identify QTLs controlling flowering and reproductive traits. They also suggested the conservation of one of the region in faba bean’s genome controlling the days of flowering also in *M. truncatula*, *L. japonicus*, *Pisum sativum*, *Cicer arietinum.* Similarly, (SNPs) from *M. truncatula* was used for the identification of QTLs [133] related to stomatal characteristics in *V. faba*. Additionally, candidate genes in the above QTLs were distinguished by synteny between the two species. The construction of highly density maps, the identification of QTLs for important traits, the use of synteny with other legumes species could be useful tools to the design of markers associated with specific traits of interest and to be used in faba bean’s breeding programs. However, despite the progress on the knowledge about faba bean’s genome the last decades, this has not been exploited on practical breeding and on cultivar release.

### 5.3. Pea (Pisum sativum L.) as a Cover Crop

*Pisum sativum* L. (Pea) is an annual cool-season grain legume (pulse) mainly cultivated for grain production and it is used in human diet. It is also used alone or in mixtures with cereals for forage production [145]. Furthermore, pea could be used as a winter or spring cover crop in temperate areas [28]. The leafy forage cultivars of pea are considered more suitable for cover crop than the grain cultivars [146]. Additionally, it has been proposed to use as bio-indicator for evaluation and monitoring the contamination and concentration of heavy metals such as cadmium and copper in soils of agroecosystems [147].

The use of pea in cover crops, as with all legume species, is related to the ability to fix atmospheric nitrogen with subsequent delivery to the soil. Its use in cover crops contributed to the increase of the available N and P for the following maize crop of a smallholder farming system in South Africa [148] as well the availability of N for following sweet corn (*Zea mays* var. saccharataSturt.) in Turkey [149]. Similar results have been obtained in the U.S. Coastal Plain and Piedmont for no-tillage corn (Zea mays L.), although pea was less efficient compared to hairy vetch (*Viciavillosa* Roth) [150].

The importance of mixtures rye (*Secalecereale*)-legumes (including pea) cover crops on organic vegetables production in California mainly due to weed control has been pointed out by [151]. In agreement with these results, weeds such as ladysthumb (*Polygonumpersicaria* L.), smooth pigweed (*Amaranthushybridus* L.), smallflowergalinsoga (*Galinsogaparviflora* Cav.) and lambsquarters (*Chenopodium album* L.) were suppressed by a mixture of rye and pea [152]. On the contrary, it was not efficiently used in a vegetable production system in Ohio due to failure to overwinter [153]. Its ability to suppress the weeds probably is related with its allelopathic activity [154]. However, the allelopathic activity could inhibit the growth of weeds but also could negatively affect the growth of the following crop especially in the case that it is planted into green residue [155].

On the other hand, its use as a cover crop reduced the wheat production about 37% compared to the control (without cover crop) on the semi-arid Canadian prairies [156]. In an organic lettuce (*Lactuca sativa* L.) production system in Turkey, it was less efficient than grain sorghum (*Sorghum bicolor*), sudangrass (*Sorghum vulgare* Pers. var.), hairy vetch (*Viciavillosa*) and grain amaranth (*Amaranthuscruentus*) [157]. It seems that its efficient use especially in the winter is related to the climatic conditions. Finally, its use as a winter cover crop in UK lower the potential of N losses but did not positively affect the yield of the following crops [158].

#### 5.3.1. Origin and Adaptation

The precise area of its origin is difficult to specify because of its early cultivation [159]. It is probably Abyssinia and Afghanistan [160]. There isarcheological evidence of its cultivation in Near East about 10,000 years B.P. [161] and Central Asia [162]. Later, it was spread in Mediterranean basin, northern and Western Europe, Africa, and Asia. Nowadays, it is among the most important annual legumes worldwide after soya bean (*Glycine max* L.), bean (*Phaseolus vulgaris* L.) and chickpea (*Ciceraerietum* L.). Russian Federation, Canada and China are the major producers [52].

It is a cool-season crop, adaptive in a wide range of environments and soil types. It grows better in cool environments with an optimum temperature around to 20 °C [163]. It also prefers well-drained, fertile soils of light texture. However, it is rather sensitive to drought, cold and salinity stresses [160].

#### 5.3.2. Genetic Diversity

*P. sativum* is a diploid (2*n* = 2× = 14) highly self-pollinated legume with a ~4063 Mb genome. There are numerous pea collections worldwide that include landraces, wild relatives, commercial cultivars and breeding lines. However, there are many duplications that finally reduce the total number of collections [164,165]. Additionally, wild *Pisum*s pecies such as *P. fulvum*, *P. sativum* ssp. *elatius*, *P. sativum* ssp. *sativum* and *P. abyssinicum* could be possible genetic resources for its breeding.

Pea’s germplasm has been evaluated by morphological and agronomic traits and extensively reviewed by [165]. Furthermore, the genetic diversity has been studied with various molecular markers including RAPDs, ISSRs, simple sequence repeat (SSRs), EST and retrotransposon-based insertion polymorphism (RBIPs) in various collections from different geographic regions [164]. According to the molecular studies, the genetic diversity of cultivars is rather low [166] and the genetic basis of some of them is very narrow. Conversely, the landraces are characterized by much more genetic diversity and could be an excellent genetic resource for pea improvement [167]. From this point of view, the evaluation and ex situ conservation of these locally adapted germplasm is of great importance.

#### 5.3.3. Important Traits for Breeding

The most important traits for breeding in relation to its use as cover crop are the N fixation and the tolerance in biotic and abiotic stresses. The biological N fixation is related to genotype [168], the rhizobia strain and their interaction [169] and is also affected by environmental factors and management practices. In particular, the reported heritability for N fixation among pea genotypes was 0.57 [168], high lighting the possibility of enhancing N fixation through breeding.

A major goal of pea’s breeding is the release of cultivars well-adapted to dry environments. The drought stress except the adverse effect on crops’ total biomass is also rapidly reduced the symbiotic nitrogen fixation (SNF) rate probably due to the reduction of photosynthesis rate [170]. Key point for a successful breeding procedure for drought tolerance in grain legumes is the using of proper screening and selection method. There are differences among the pea’s cultivars and germplasm in their response to drought stress [171]. In particular, for pea, the genotypes (*af*) (semi-leafless), with leaflets transformed into tendrils, performed better under water-deficit conditions compared to normal-leafed genotypes [172]. Furthermore, Sanchez et al. reported that cultivars that better maintained the turgor potential and osmotic adjustment were more drought-tolerant [173]. Recently, the breeding programs have been focused on the selection of early flowering genotypes to use in dry and hot areas in combination with proper management practices [171].

Cold tolerance is also an important trait especially for the winter cover crops. Pea is less cold tolerant than the other grain legumes [174]. Cold tolerance is a quantitatively inherited trait [175]. Among the pea’s cultivars only the forage type (*P. sativum* ssp. *arvense*) are referred as cold tolerant [163] and as consequent are the basic genetic resources in the winter pea breeding programs. Additionally, the accessions from cold regions had higher cold tolerance than the others in an evaluation of a large-scale germplasm collection of 3672 accessions during winter in field conditions in Chine [176]. The cold tolerance mainly contributed to the delayed initiation of flowering which is the most sensitive growth stage [177]. Particularly in pea, the flowering locus *Hr* is probably related with the cold tolerance [178]. The progeny test from the crosses between tolerant and sensitive cultivars evidenced that it is possible the transfer of cold tolerance from the first to latter [160].

Regarding the biotic stresses, fungi (*Ascochyta complex*, *Peronospora viciae*, *Erysiphe* spp. *Fusarium* spp.), virus such as pea seed-borne mosaic virus (PSbMV), pea enation mosaic virus (PEMV) and bacteria (*Pseudomonas*) are the major threats for its crops [160,163]. Additionally, the insect pests as pea leaf weevil (*Sitona lineatus* L.) and seed weevil (*Bruchus pisorum*) have an adverse effect on pea’s crops [163]. Differentiation among cultivars in terms of susceptibly to fungi has been reported for *Ascochyta complex* [179] and *Erysiphe* spp. [180]. *Erysiphe* spp. is related to three major genes (*Er1*, *Er2* and *Er3*) [164]. The recessive er1 contribute to the cultivars’ resistance [181] and it has been used in breeding programs. There is evidence that the forage cultivars are less susceptible to fungi infection than the edible ones [182]. It is noteworthy that in all cases only moderate resistance to biotic stresses in pea cultivars has been reported [183]. This means that for an efficient breeding procedure the evaluation and exploitation of a broader gene pool in combination with the use of molecular tools is required.

The evaluation of the existing collections is a key factor for their breeding programs. The adopted conventional breeding methods are similar to those used for other self-pollinated species and include controlled hybridization and selection [163]. The selection methods usually are combination of bulk, pedigree, backcross and single-seed descent methods [184]. Generally, the germplasm of wild relatives is the less representative in the existing collections [185] indicating their limited use as gene pool in the breeding programs. In this regard, *P. fulvum* could be an excellent donor for biotic stresses resistance genes [165]. The broadening of cultivated germplasm is of great importance for pea’s modern breeding programs. Candidate gene pools could be the wild relatives such as *P. fulvum* and *P. sativum* ssp. *elatius*. The wild relatives are a valuable gene pool that it should be evaluated and conserved before it is lost.

#### 5.3.4. Molecular Approach for Breeding

The use of the molecular tools in pea breeding programs could increase their efficiency and accelerate their progress. However, the progress in genomics of pea has fallen behind compared to other important crops mainly because of its genome size and the number of repetitive elements [186]. Nevertheless, there are available data, including **b**acterial **a**rtificial **c**hromosome (BAC) libraries, molecular markers, transcriptomes and proteomes, which are extensively reviewed by [164].

Most studies focus on disease resistance; particularly, four QTLs for resistance to *Orobanche crenata* Forsk, and two quantitative trait loci (QTL) for resistance to *Pseudomonas syringae* pv. *Syringae* was identified by Fondevilla et al. [187,188]*.* Furthermore, three novel QTLs for resistance to *Mycosphaerella pinodes*, two for resistance to *Orobanche crenata* and one for root length were identified using SSRs [189]. The development of a SCAR marker related with er1 gene for resistance to powdery mildew was studied by [190]. Additionally, the high resolution melt (HRM) marker er1-5/HRM54 could be suitable for large-scale breeding programs for resistance to powdery mildew (*Erysiphe pisi* D.C.) [191].

It seems likely that progress in genomics research for pea is rapid. A linkage map in Chinese accessions contained 199 SSR markers was constructed and the use of marker-assisted selection in pea breeding in the near future is anticipated [192,193]. A single-nucleotide polymorphism (SNP) marker platform using next-generation transcriptome sequencing technology of eight diverse *Pisum* accessions was developed by Sindhu et al. [194]. Additionally, they established syntenic relationships of this map with the corresponding ones of *Medicago truncatula* and *Lens culinaris* Medik. The similar technology was also used by Boutet et al. [195] for sequencing of four pea lines. All these data could be used in both mapping and marked-assisted selection. However, despite the progress in genomics of *P. sativum*, there is still limited availability in molecular resources compared to other crops.

## 6. Conclusions

The key utilization characteristics of grain legumes uses as cover crops are the following.

1. To fully exploit their benefits, proper integration into agro-ecosystems should be safeguarded. In this context, the interaction of the effects with various components such as soil types, wet or dry growing conditions, or lodging should be validated.
2. Different roles for major grain legumes have been attributed. Cowpea could be an excellent summer cover crop that contributes to the weed suppression, reduction of soil erosion and increase of soil N. On the other hand, the importance of faba bean, as winter cover crop, related to its ability of N fixation has been monitored. Finally, peas are related to their enhanced ability to compete with weeds.
3. Generally, high level of genetic diversity exists in the germplasm of the aforementioned species.
4. Recent developments into whole genome sequencing (e.g., cowpeas) would allow significant contributions into crop improvements.
5. Despite the progress in breeding programs, the existing genetic diversity and molecular tools have not been completely exploited.

## 7. Prospects for Future Research

The most important future research trends are as follows:

1. Sound empirical data (from field trials) are needed to quantify the positive effects of legumes on major agronomic characteristics such as yields, weed competition, nitrogen use, P and K availability and general crop health. For example, clear differences of its effects on the succeeding crops should be manifested as related to sowing time (autumn vs. spring sowing times).
2. Grain legume crop improvements would be documented in the near future. Those crop improvements would be based upon gene expression information and other resources that are rapidly accumulated. In cowpeas, for example, breeding programs have been starting to exploit these resources for molecular breeding, especially for MARS and MABC. The new publicly available resources and knowledge would help the breeding of cowpea in order to become an ideal summer cover crop. In the post genomic era, the ultimate achievement for a breeder will be the simple detection of the chromosome sections that are inherited from each parent in order to assist the selection procedure and diminish the need for widespread field trials.

## Figures and Tables

**Table 1:** The desirable traits that cowpea, faba bean and winter pea obtain when used as cover crops along with the most important categories of crops that are benefited according to the literature.

*Cover Crop Desirable Traits*	Cowpea	Faba Bean	Winter Pea
Source of Nitrogen	+	+++	+
Reduction of N leaching	+	+	+
Reduction of soil erosion	+++	+	+
Improvement of soil fertility	+++	+++	+
Weed management	+++	+	+++
Winter hardiness	−	+++	+
Drought resistance	+++	−	−
Rapid growth	+++	+	+
*Crops to be benefited*			
Cereals	+	+	+++
Vegetables	+	+	+
Organic farming	+++	+	+
Vineyard	na	+++	na

Excellent: +++, Moderate: +, Low: −, Not Available Information: na.
